# The HSP GRP94 interacts with macrophage intracellular complement C3 and impacts M2 profile during ER stress

**DOI:** 10.1038/s41419-020-03288-x

**Published:** 2021-01-22

**Authors:** Killian Chaumonnot, Sophie Masson, Hugo Sikner, Alexanne Bouchard, Valentin Baverel, Pierre-Simon Bellaye, Bertrand Collin, Carmen Garrido, Evelyne Kohli

**Affiliations:** 1 UMR INSERM/uB/AGROSUP 1231, Team 3 HSP-Pathies, labellisée Ligue Nationale contre le Cancer and Laboratoire d’Excellence LipSTIC, Dijon, France; 2 UFR des Sciences de Santé, Université de Bourgogne, Dijon, France; 3 Centre anti-cancéreux Georges François Leclerc, Dijon, France; 4 UMR uB/CNRS 6302, Institut de Chimie Moléculaire, Dijon, France; 5 CHU, Dijon, France

**Keywords:** Immunology, Innate immune cells

## Abstract

The role of GRP94, an endoplasmic reticulum (ER) stress protein with both pro- and anti-inflammatory functions, has not been investigated in macrophages during ER stress, whereas ER stress has been reported in many diseases involving macrophages. In this work, we studied GRP94 in M1/LPS + IFNγ and M2/IL-4 primary macrophages derived from human monocytes (isolated from buffy coats), in basal and ER stress conditions induced by thapsigargin (Tg), an inducer of ER calcium depletion and tunicamycin (Tm), an inhibitor of N-glycosylation. We found that GRP94 was expressed on the membrane of M2 but not M1 macrophages. In M2, Tg, but not Tm, while decreased GRP94 content in the membrane, it induced its secretion. This correlated with the induction of a pro-inflammatory profile, which was dependent on the UPR IRE1α arm activation and on a functional GRP94. As we previously reported that GRP94 associated with complement C3 at the extracellular level, we analyzed C3 and confirmed GRP94-C3 interaction in our experimental model. Further, Tg increased this interaction and, in these conditions, C3b and cathepsin L were detected in the extracellular medium where GRP94 co-immunoprecipitated with C3 and C3b. Finally, we showed that the C3b inactivated fragment, iC3b, only present on non-stressed M2, depended on functional GRP94, making both GRP94 and iC3b potential markers of M2 cells. In conclusion, our results show that GRP94 is co-secreted with C3 under ER stress conditions which may facilitate its cleavage by cathepsin L, thus contributing to the pro-inflammatory profile observed in stressed M2 macrophages.

## Introduction

Glucose-regulated protein 94 (GRP94, also known as Gp96) is an endoplasmic reticulum (ER) member of the heat shock protein HSP90 family whose expression is increased during ER stress^1^. GRP94 has also been reported in physiological or pathological situations in the extracellular medium^2–4^ and at the surface of tumor cells (reviewed in ref. ^5^). Intracellular GRP94 modulates inflammatory and immune responses as it acts as a molecular chaperone for most TLRs and integrins^6,7^. On the other hand, it controls regulatory T cells by chaperoning the docking receptor for TGF-β, GARP (Glycoprotein A repetitions predominant)^8^. Also, GRP94 has been shown to be associated with gut microbiota immune tolerance, its expression being strongly induced during monocyte differentiation into intestinal macrophages in healthy patients but not in patients with Crohn’s disease^9^. In addition, Hua et al.^10^ have reported that GRP94 was essential for dendritic CD11c cells to maintain gut tolerance. However, how GRP94 induces tolerance in macrophages has not been deciphered.

Macrophages are diverse and, depending on the microenvironment, different types are induced in vivo. The classical pro-inflammatory (M1) and alternatively activated (M2) macrophage phenotypes are considered to be at the extremes of this spectrum^11^. Although ER stress has been reported in many diseases involving macrophages (obesity, atherosclerosis, neuroinflammation, arthritis, cancer, pulmonary fibrosis…), its impact on macrophage polarization remains unclear. Indeed, some authors report the induction or amplification of pro-inflammatory M1 type cytokines by ER stress^12,13^ or an augmented M2 polarization of adipose tissue macrophages after blocking IRE1α^14^ whereas a M2-like profile was observed in stressed tumor associated macrophages^15,16^ and, in lung macrophages, ER stress appears to favor the M2 phenotype with profibrotic effects^17^.

To better understand GRP94 role in macrophages, we analyzed intracellular and cell surface expression of GRP94 in M1 and M2 macrophages derived from PBMC of healthy volunteers, at basal and ER stress conditions. Moreover, as we previously showed that extracellular GRP94 associates with the complement C3^4^ and Liszewski et al. have reported that shuttling of the intracellular C3-activation-system to the cell surface upon T cell stimulation induces autocrine pro-inflammatory cytokine production^18^, we analyzed the interaction GRP94-C3 in macrophages and studied its potential role in their polarization.

## Results

### GRP94 cell membrane expression in M2(IL-4) macrophages is modified by ER stress

Macrophages were differentiated from PBMC of healthy volunteers and activated into pro-inflammatory M1 or alternatively activated M2 macrophages^19^. ER stress was induced during activation using either thapsigargin (Tg), an inducer of ER calcium depletion, or tunicamycin (Tm), an inhibitor of N-glycosylation, at concentrations reported to induce mild ER stress^20^.

**Fig. 1:**
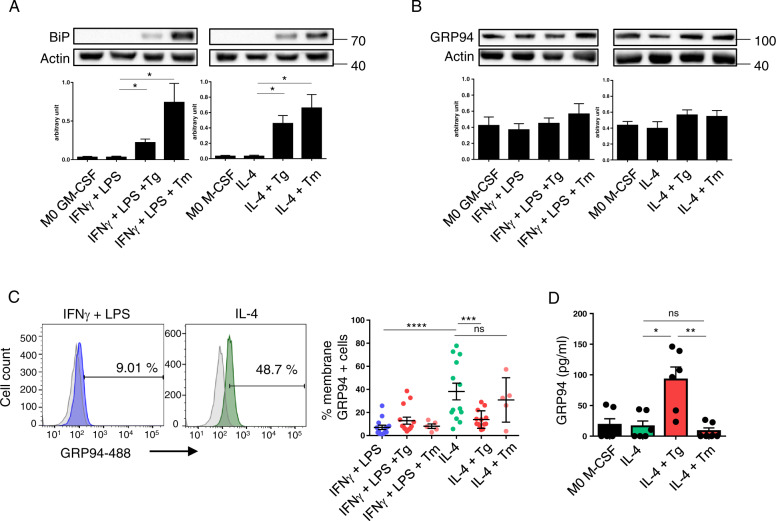
GRP94 expression by M1 (IFNγ-LPS) and M2(IL-4) macrophages treated or not with Tg or Tm. M1 and M2 macrophages derived from PBMC of healthy volunteers were activated in the presence of GM-CSF and M-CSF, respectively. Tg (25 nM) or Tm (100 nM) was added to cell cultures during all the activation period. **A**, **B** Western blot analysis (*n* = 4) of BiP (**A**) and GRP94 (**B**) in M1 and M2 activated macrophages (24 h) treated or not with Tg or Tm. **C** Left panel, GRP94 membrane expression on 48 h activated M1 and M2 analyzed by FACS; Right panel, histograms of GRP94 membrane expression in M1 and M2 macrophages under basal or Tg or Tm conditions (*n* = 14 donors except for Tm *n* = 5). **D** GRP94 quantification by ELISA in macrophages M2 cell culture supernatants activated for 48 h and treated or not with Tg or Tm (*n* = 6).

We first analyzed BiP expression and showed an increase in M1 and M2 macrophages after treatment with Tg and Tm (Fig. 1A). Then, we analyzed intracellular and membrane GRP94. Intracellular GRP94 did not differ between M1 and M2 and despite a trend to an increase at 24 h after Tg and/or Tm treatment, it was not significantly modified (Fig. 1B) nor was it at any earlier time-point tested (Supplementary Fig. S1A). This result may be explained by the low concentrations of Tg and Tm used. GRP94 was expressed at the membrane of M2 but not M1 macrophages (Fig. 1C and Supplementary Fig. S1B). Moreover, it decreased after Tg but not Tm exposure (Fig. 1C, *p* = 0.005 and 0.8122 at 48 h post-treatment, respectively; Supplementary Fig. S1C at 6, 12 and 24 h).

We next quantified GRP94 in the supernatants and found that Tg (but not Tm) induced GRP94 secretion (*p* = 0.0148) by M2 (Fig. 1D).

**Fig. 2:**
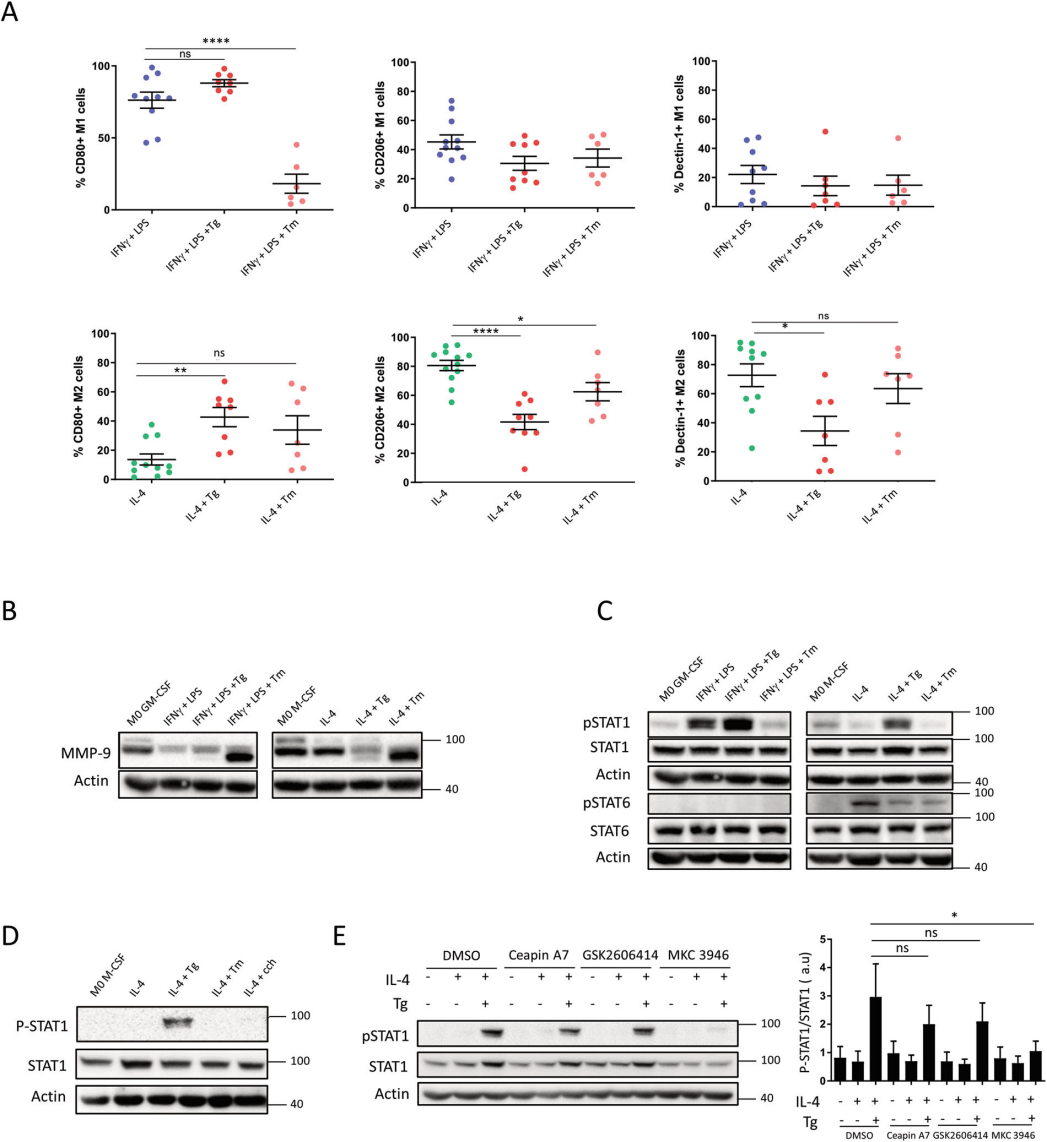
Tg impacts M2(IL-4) phenotype *via* the UPR IRE1α pathway. **A** Membrane expression by FACS analysis of CD80, CD206 and Dectin-1 on M1 and M2 macrophages treated or not with Tg or Tm (*n* = 6–9). **B** Western-blot analysis of MMP-9 (*n* = 3) in M1 and M2 macrophages treated or not with Tg or Tm during 24 h. **C** Western-blot analysis of signalization proteins pSTAT1/STAT1 and pSTAT6/STAT6 (*n* = 5) in 24 h activated M1 and M2 macrophages treated or not with Tg or Tm. **D** pSTAT1/STAT1 signalization proteins in 24 h activated M2 macrophages treated or not with Tg, Tm or carbachol (cch, 1 μM) (*n* = 3). **E** Western-blot analysis of signalization proteins pSTAT1/STAT1 in 24 h activated M2 macrophages treated or not with Tg and with or without 5 μM of ceapin A7, GSK2606414 and MKC 3946, inhibitors of the ATF6, PERK and IRE1α UPR pathway respectively (*n* = 4). *p < 0.05; **p < 0.01; ***p < 0.005; ****p < 0.001).

To evaluate Tg and Tm impact on macrophage phenotype, we analyzed CD80 and CD206 and dectin-1 as M1 and M2 markers, respectively. Tg had no impact on M1 whereas it increased CD80 (*p* = 0.0075) and decreased CD206 and dectin-1 on M2 (*p* < 0.0001 and *p* = 0.0224, respectively) (Fig. 2A), suggesting the induction of a switch to a pro-inflammatory phenotype. In accordance with our previous results, Tm had a less clear effect. While it decreased CD80 on M1 (*p* = < 0.0001), it had hardly any effect on M2 markers compared to Tg. The active form of MMP9 was highly expressed in M2 cells as expected, whereas, in ER stress conditions, we again observed a difference between Tg and Tm as only Tg decreased MMP9 expression (Fig. 2B). Of note, Tm had an impact on MMP9 migration that was attributed to decreased N-glycosylation^21^.

We also analyzed pSTAT1 and pSTAT6^22^. In accordance with the increase in CD80 and the decrease in CD206 and dectin-1, pSTAT1 increased in Tg-treated M2 whereas pSTAT6 decreased (Fig. 2C). In M1, the only change was a weak increase in pSTAT1. In contrast, Tm induced a major decrease in pSTAT1 in M1, correlating with the decrease in CD80 and, although it decreased pSTAT6 in M2 cells, it did not induce pSTAT1.

As differences between Tg and Tm could correlate with differences in ER stress induction, we analyzed cell death and the activation of the 3 UPR pathways. Tg and Tm, at the concentrations used, did not induce significant cell death at 72 h (Supplementary Fig. S2A). Concerning the UPR, Tg and Tm induced IRE1α and PERK activation in M1 and M2 but this effect was more important for Tg (Supplementary Fig. S2B). This difference was particularly marked in M2 at 24 h (Supplementary Fig. S2C). The cleaved fragment resulting from ATF6 activation was not observed at any time point after Tg or Tm treatment (data not shown), suggesting that ATF6 was not activated in our model. We also showed that carbachol (Cch), an agonist of ER IP3 receptor which leads to the release of calcium from the ER into the cytosol, did not induce IRE1α or PERK activation (Supplementary Fig. S2C) nor pSTAT1 or CD206-CD80 changes in M2 macrophages (Fig. 2D and Supplementary Fig. S2D), thus confirming that the UPR is necessary for the pro-inflammatory profile observed in M2 after Tg treatment. Finally, using inhibitors of the three pathways, we showed that MK-3946, an inhibitor of pIRE1α, significantly decreased pSTAT1 expression (*p* = 0.0185) in Tg-treated M2 macrophages (Fig. 2E).

These results show that GRP94 is expressed at the membrane of M2(IL-4) but not M1(IFNγ-LPS) human primary macrophages differentiated and activated in vitro. Moreover, in M2, Tg downregulates membrane GRP94 and this is associated with its secretion and a switch to a pro-inflammatory profile, which correlates with the IRE1α pathway activation.

### Functional GRP94 is necessary for the modulation of M2 towards the pro-inflammatory profile induced by Tg

To study the role of GRP94 in M2, we used PU-WS13^23^, a selective inhibitor which inhibits GRP94 in its closed form, with the client protein bound to it. PU-WS13 was added 24 h before IFNγ + LPS or IL-4 and Tg, so that GRP94 was inhibited before and during the activation period.

We first showed that PU-WS13 did not induce ER stress as shown by the lack of BiP increase (Supplementary Fig. S3A) and was not cytotoxic after 72 h of treatment at the concentrations tested (Supplementary Fig. S3B).

**Fig. 3:**
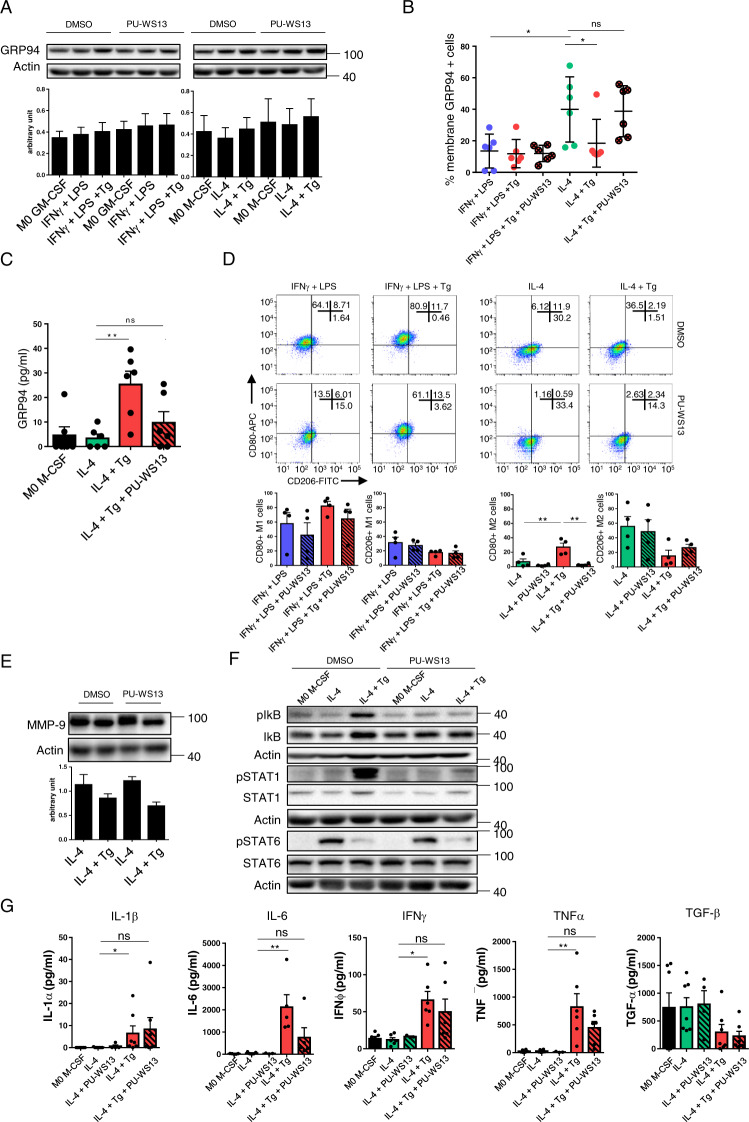
Inhibition of GRP94 by PU-WS13 impairs the effect of Tg-induced ER stress on M2 macrophages. M1 and M2 macrophages from PBMC healthy volunteers were activated in the presence of GM-CSF and M-CSF, respectively. Tg was added to cell cultures during the 48 h activation period. PU-WS13 or DMSO as vehicle was added in cell culture medium at the concentration of 25 μM 24 h before and during all the activation period. **A** Intracellular GRP94 western-blot analysis (*n* = 3). **B** FACS analysis of membrane GRP94 expression (*n* = 6). **C** GRP94 quantification by ELISA in activated M2 cell culture supernatants (*n* = 6). **D** CD80 and CD206 analysis by flow cytometry (*n* = 4). **E**, **F** Western blot analysis of MMP9 in activated M2 macrophages (*n* = 4) (**E**) and of the signalization proteins pSTAT1/STAT1, pSTAT6/STAT6 and pIκB/ IκB (*n* = 4) (**F**). **G** Analysis of pro- and anti-inflammatory cytokines in M2 cell culture supernatants (*n* = 5–7). IL-1β, IL-6, IFNγ and TNFα were quantified by the Multiplex™ method and TGF-β was quantified by ELISA (*p < 0.05; **p < 0.01; ***p < 0.005; ****p < 0.001).

Then, we analyzed intracellular and membrane GRP94. As expected^24^, PU-WS13 did not affect intracellular GRP94 (Fig. 3A). Concerning membrane GRP94, PU-WS13 had no impact on Tg-treated M1. On M2, whereas PU-WS13 had no effect on its own (Supplementary Fig. S3C), it clearly prevented membrane GRP94 decrease induced by Tg (Fig. 3B) as well as its secretion (Fig. 3C), finding again an inverse correlation between membrane-bound and secreted GRP94.

We also analyzed CD206 and CD80 expression and showed that PU-WS13 at the concentration of 25 μM prevented both the increase in CD80 and the decrease in CD206 in Tg-treated M2 (Fig. 3D). PU-WS13 did not induce any change in these markers in M2 in the absence of Tg, or in M1, treated or not with Tg. A dose-dependent effect analysis showed that PU-WS13 partially prevented CD80 increase at 1 μM whereas the effect on CD206 required higher concentrations (Supplementary Fig. S3D). We also analyzed the effect of NVP-BEP800, a selective inhibitor of HSP90 and did not observe any change in CD80 and CD206 expression at the concentrations of 50 and 500 nM (Supplementary Fig. S3D), higher concentrations being toxic (Supplementary Fig. S3E). This confirms a specificity of PU-WS13 for GRP94^23^.

We therefore focused on M2 to analyze MMP9 expression and STAT1, IkB and STAT6 phosphorylation. PU-WS13 did not prevent the decrease in MMP9 (Fig. 3E) nor the decrease in pSTAT6 induced by Tg (Fig. 3F) but prevented Tg-induced increase in pSTAT1 and pIKB.

We then analyzed cytokines in the supernatants and showed that, conversely to untreated M2, Tg-treated M2 produced IFNγ, IL-6 and TNFα as well as low but significant levels of IL-1β, whereas TGF-β was decreased (Fig. 3G), confirming the pro-inflammatory profile induced by Tg in M2. PU-WS13 decreased IFNγ, IL-6 and TNFα secretion, but did not prevent the decrease in TGF-β, in accordance with its absence of effect on STAT6 phosphorylation (Fig. 3F).

These results show that functional GRP94 is necessary for the induction of the pro-inflammatory profile induced by Tg in M2 macrophages, in the absence of exogenous inducers such as LPS or IFNγ. In these conditions, GRP94 may contribute to fine-tune the balance pro/anti-inflammatory profile in M2 macrophages.

### Tg decreases CD11b expression in M2 macrophages

GRP94 chaperones pro- and anti-inflammatory proteins, among them TLR4, GARP and the integrin α and β chains that constitute the CR3 and CR4 receptors for the inactivated iC3b fragment of C3, which has been shown to induce tolerance in macrophages^25^. Those proteins may be involved in the modulation of the M2 profile observed after Tg treatment.

**Fig. 4:**
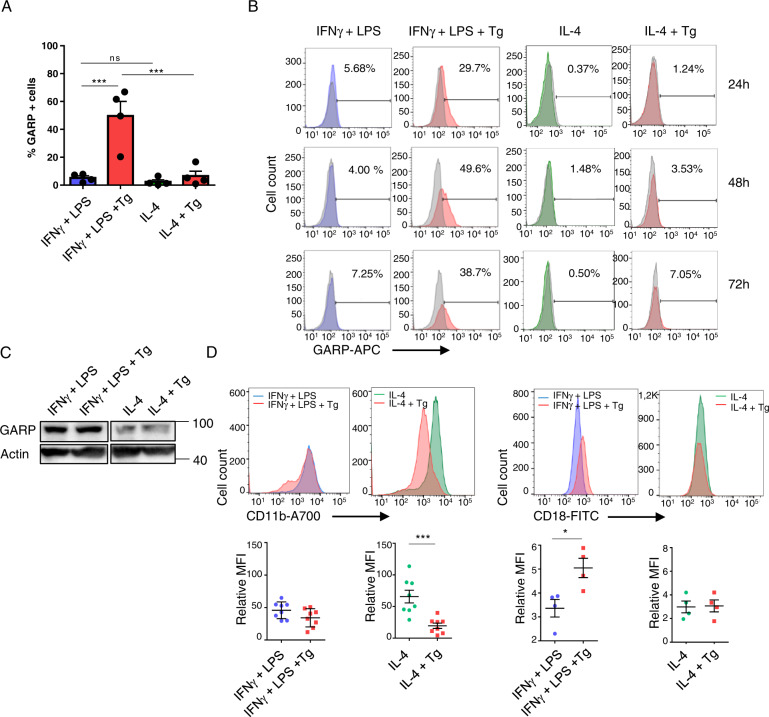
GARP and CD11b expression on M1 and M2 macrophages. **A** FACS analysis of GARP membrane expression on M1 and M2 macrophages treated or not with Tg (*n* = 4). **B** Kinetics of GARP expression on M1 and M2 macrophages treated or not with Tg for the indicated times (*n* = 3). **C** GARP analysis by western-blot in M1 and M2 macrophages treated or not with Tg (a representative image is shown; *n* = 2). **D** CD11b (*n* = 8) and CD18 (*n* = 4) membrane expression on M1 and M2 macrophages treated or not with Tg (*p < 0.05; **p < 0.01; ***p < 0.005; ****p < 0.001).

The results shown in Fig. 4A demonstrate that M2 (treated or not with Tg) did not express membrane GARP, indicating that it might not be responsible for the lack of recover of the anti-inflammatory profile observed after treatment with PU-WS13. Analysis of membrane GARP after 24 h, 48 h, and 72 h of Tg treatment (Fig. 4B), as well as of total GARP at 48 h by western-blot (Fig. 4C) confirmed this result. In contrast, GARP was expressed in M1 and, after Tg treatment, a subset of them expressed GARP (*p* = 0.0006). GARP expression on M1 was significantly decreased after PU-WS13 treatment (Supplementary Fig. S4).

We then analyzed CD11b, the integrin alpha M chain of CR3, and CD18, the integrin beta 2 chain common to CR3 and CR4, both receptors for iC3b, and found a decrease in CD11b mean fluorescence intensity after Tg treatment in M2 but not M1 macrophages (*p* = 0.0009) (Fig. 4D).

Based on these results and on our previous results showing that extracellular GRP94 interacts with the complement C3^4^, we decided to study the intracellular interaction GRP94-C3 in macrophages.

### GRP94 interacts with intracellular C3 in macrophages

**Fig. 5:**
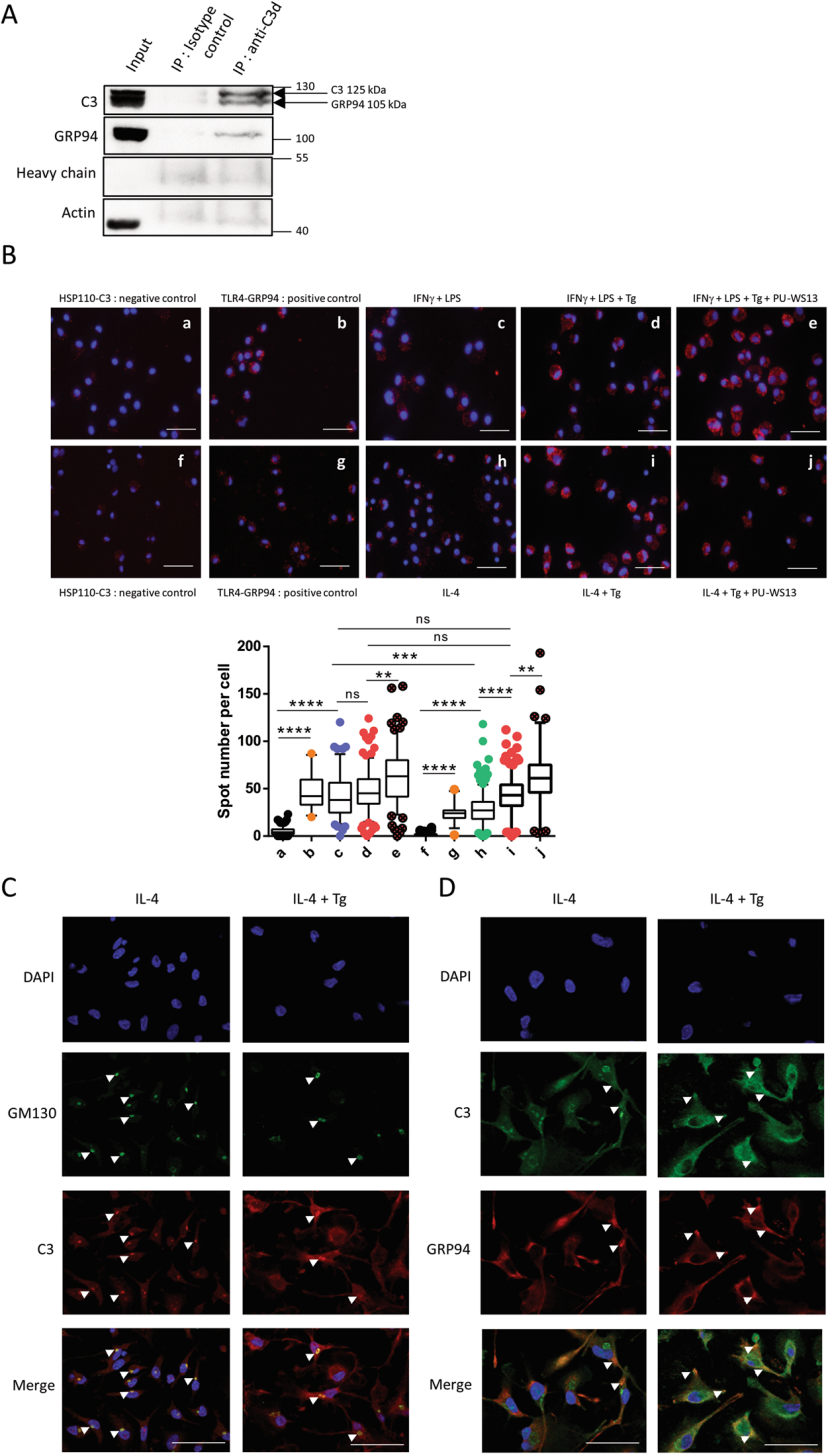
GRP94-C3 interaction in THP1 cells and in M1 and M2 macrophages derived from monocytes of healthy donors. **A** Immunoblot of C3 and GRP94 co-immunoprecipitated in THP1 cells using an anti-C3d polyclonal antibody (*n* = 3). GRP4 was revealed first then C3, without previous stripping. **B** Staining and quantification of proximity ligation assay (Duolink™PLA) between C3/C3b and GRP94 in activated M1 and M2 macrophages treated or not with Tg and pretreated or not with PU-WS13 or DMSO as vehicle (*n* = 3); the box plot represents the number of spots for each cell analyzed. The boundary of the box closest to zero indicates the 25th percentile, the black line within the box marks the median and the boundary of the box farthest from zero the 75th percentile. Whiskers below and above the box indicate the 5th and 95th percentiles. Points below and above the whiskers indicate outliers outside the 5th and 95th percentiles (*p < 0.05; **p < 0.01; ***p < 0.005; ****p < 0.001). **C** Immunofluorescence staining of C3/C3b and GM130 in activated M2 macrophages treated or not with Tg (*n* = 3, representative images). **D** Immunofluorescence staining of GRP94 and C3/C3b (*n* = 3, representative images). White arrows indicate overlapping areas for each labeling. Scale bar= 20 μm.

To study GRP94-C3 association, we first used THP1 cells to co-immunoprecipitate GRP94 and C3/C3b. GRP94 and the alpha chain of C3 co-immunoprecipitated as shown in Fig. 5A.

Then, we used the proximity ligation assay (Duolink™ PLA) and confirmed the GRP94-C3/C3b interaction in M1 and M2 macrophages (Fig. 5B). In M1, quantification of the GRP94-C3 interaction was similar to that of GRP94-TLR4, used here as a positive control^7^. In M2, the number of interactions was lower compared to M1 (*p* = 0.0003) and increased after Tg treatment, suggesting that Tg favored the interaction GRP94-C3/C3b. Finally, the interaction in Tg-treated M1 and M2 was further increased in the presence of PU-WS13 (*p* = 0.0038 and *p* = 0.0033 respectively). This sequestration of C3 by the complex GRP94/PU-WS13 is explained by the fact that PU-WS13 maintains the chaperone in its closed form^24^.

We next analyzed GRP94 and C3/C3b by immunofluorescence. Since GRP94 and C3 are secreted, we also used GM130, a Golgi apparatus marker. As shown in Fig. 5C, in M2, C3/C3b colocalized with GM130 but not or hardly with GRP94 (Fig. 5D). In Tg-treated M2 cells, colocalization of GRP94 and C3 was clearly observed; C3/C3b still colocalized with GM130, but had a more diffuse staining and GRP94 showed an extensive overlap in the cytosolic regions highly stained for C3/C3b, especially at the tip of pseudopods. These results show that Tg may favor GRP94 and C3/C3b colocalization, in accordance with our PLA results.

### The balance iC3b/C3b differs between untreated and Tg-treated M2 macrophages

**Fig. 6:**
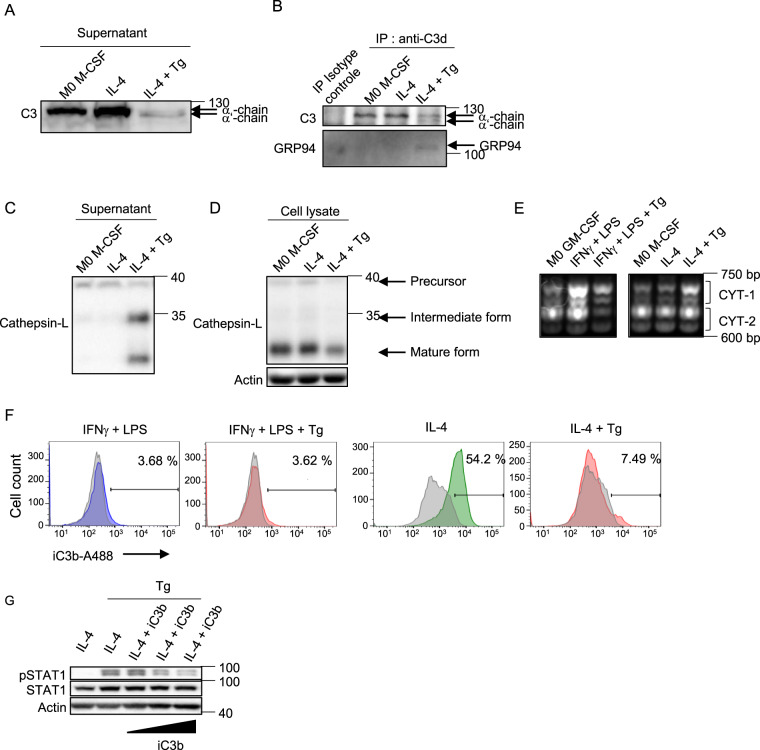
C3b/iC3b balance in untreated and Tg-treated M2 macrophages. **A** Western-blot analysis of C3/C3b in cell culture supernatants of 48 h activated M2 macrophages treated or not with Tg (*n* = 3). **B** Co-immunoprecipitation of C3/C3b and GRP94 in M2 macrophages supernatants using an anti-C3d polyclonal antibody (*n* = 2). Western-blot analysis of cathepsin L both in cell supernatants (*n* = 3) (**C**) and in cell lysates (**D**) of 48 h activated M2 macrophages treated or not with Tg (*n* = 3). **E** CYT1 and CYT2 CD46 isoforms in M1 and M2 macrophages treated or not with Tg were analyzed by PCR (*n* = 3). **F** iC3b membrane- bound FACS analysis on M1 and M2 macrophages treated or not with Tg (*n* = 3). **G** Western-blot analysis of pSTAT1/STAT1 signalization proteins in M1 macrophages treated with Tg in the presence or absence of increasing amounts of recombinant iC3b (10; 100; 1000 nM) (*n* = 3).

As GRP94 was secreted in Tg-treated M2 supernatants and colocalized with C3/C3b, we analyzed C3/C3b in the supernatants. The α−chain of C3 was detected in all conditions (MW 125 Kd), although at much lower levels in Tg- treated conditions, whereas the α‘-chain (C3b, MW 115 Kd) was only detectable in the Tg-treated cells supernatants (Fig. 6A). We next performed C3/C3b and GRP94 co-immunoprecipitation experiments in the supernatants and confirmed these results. Interestingly, only in those Tg-treated supernatants GRP94-C3-C3b interaction could be detected (Fig. 6B).

We then analyzed cellular and secreted cathepsin L, the protease described to be responsible for the cleavage of C3 into C3b and C3a in T cells^18^. The results showed the presence of cathepsin L only in Tg-treated M2 supernatants (Fig. 6C) which correlated with its decrease in cell lysates (Fig. 6D).

CD46 being both a cofactor of factor I that cleaves C3b into iC3b and a receptor of C3b displaying CYT1 and CYT2 isoforms that are involved in T cell differentiation, we analyzed CD46 CYT1 and CYT2 isoforms mRNA. CYT1 and CYT2 isoforms were differently expressed in M1 and M2 (Fig. 6E), M2 expressing mainly CYT2 isoforms. In contrast, Tg treatment induced an increase in the ratio CYT1/CYT2 in both phenotypes.

We finally analyzed membrane-bound iC3b and found that it was present on M2 but not M1 macrophages and strongly decreased after Tg treatment (Fig. 6F). Then, we used recombinant iC3b to see if it could modulate Tg-induced pro-inflammatory phenotype in M2. iC3b was added at the same time as IL-4 and Tg and we used as readout pSTAT1 because it was strongly affected by Tg (Fig. 2C). As shown for the GRP94 inhibitor PU-WS13 (Fig. 3F), iC3b blocked Tg-induced increase in pSTAT1 in a dose-dependent manner (Fig. 6G).

These results suggest that C3b/iC3b may play a role in the modulation of the pro-inflammatory profile observed in Tg-treated M2, in adequacy with the ratio CYT1/CYT2 CD46 isoforms.

### GRP94 affects membrane-bound iC3b and intracellular cathepsin L

**Fig. 7:**
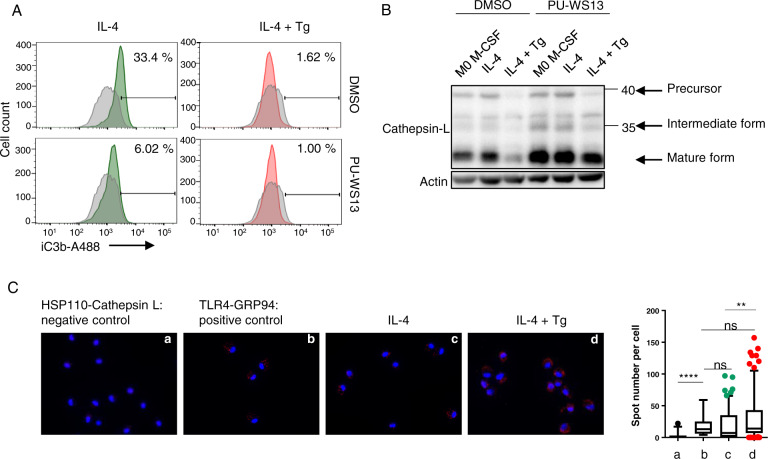
GRP94 affects membrane-bound iC3b and intracellular cathepsin L. **A** FACS analysis of iC3b membrane-bound on 48 h activated M2 macrophages treated or not with Tg, in the presence or absence of PU-WS13 (*n* = 4). **B** Western-blot analysis of cathepsin-L in lysates from M2 macrophages treated or not with Tg and pretreated or not with PU-WS13 (*n* = 4). **C** Staining and quantification of proximity ligation assay (Duolink™PLA) between cathepsin L and GRP94 in M2 macrophages treated or not with Tg (*n* = 4) (*p < 0.05; **p < 0.01; ***p < 0.005; ****p < 0.001).

We next analyzed whether PU-WS13 affected membrane-bound iC3b. As shown in Fig. 7A, PU-WS13 decreased membrane-bound iC3b on M2, suggesting the involvement of GRP94. Then, we analyzed the effect of PU-WS13 on cathepsin L and observed that, while in untreated macrophages it tended to increase intracellular cathepsin L, PU-WS13 clearly prevented Tg-induced cathepsin L decrease (Fig. 7B). Finally, we analyzed the interaction between intracellular GRP94 and cathepsin L using PLA and showed a proximity between GRP94 and cathepsin L that increased after Tg treatment (Fig. 7C).

We propose that GRP94, which is secreted in Tg-treated macrophages together with C3 and cathepsin L in the extracellular medium, may allow the activation of C3 by cathepsin L correlating with the induction of the pro-inflammatory profile observed.

## Discussion

We showed that GRP94 is expressed at the membrane of M2 but not M1 macrophages. Moreover, the ER stress inducer thapsigargin, while decreasing GRP94 membrane expression on M2, induced GRP94 secretion together with a pro-inflammatory profile. These results suggest an inverse correlation between membrane and secreted GRP94, membrane GRP94 being associated with the anti-inflammatory profile of M2, whereas the lack of membrane expression is associated with a pro-inflammatory profile. This GRP94-dependent pro-inflammatory effect, observed with Tg but not Tm at the concentrations used, was associated with the IRE1α pathway of UPR activation. Our results are in line with published data demonstrating that GRP94 secretion occurs after Tg but not Tm treatment^26,27^. Moreover, they are in adequation with studies showing a link between IRE1α and a switch to a pro-inflammatory profile in murine M2 macrophages^14,28^.

Since Tg is an inhibitor of the ER Ca + + ATPase, the Tg-GRP94-dependent effect on macrophages may be related to ER calcium balance. Indeed, an intracellular increase in calcium from the ER and/or the extracellular space has been reported in TLR activation and inflammation in macrophages^29–32^ and in macrophage polarization^33^. However, the lack of effect observed with carbachol alone, an agonist of ER membrane IP3R receptor, indicated that it is the UPR that is indispensable for the Tg-induced effect on M2 macrophages.

To our knowledge, this is the first report demonstrating the presence of GRP94 at the cell surface of M2 macrophages. Interestingly, we also found the association of GRP94 with M2 in vivo. We have analyzed CD206 + macrophages infiltrating murine triple negative breast cancer 4T1 biopsies and found that GRP94 was present on their membrane (Fig. S5). We propose membrane GRP94 may be a signature of M2(IL-4) cells.

To better understand the role of GRP94 in M2, we used its specific inhibitor, PU-WS13^24^. PU-WS13 did not affect M2 markers nor TGF-β levels, suggesting that in our conditions, functional GRP94 may not be essential for maintaining the M2 phenotype at basal conditions. In contrast, PU-WS13 had a striking effect on Tg-treated M2 by preventing Tg-induced GRP94 secretion and pro-inflammatory markers increase. We concluded that secreted GRP94 may play a pro-inflammatory role. However, while PU-WS13 inhibited Tg-induced pSTAT1 expression, it did not allow to restore pSTAT6 nor the anti-inflammatory markers of the M2 profile. The effect of GRP94 in M2 could not involve its client protein GARP, which activates TGF-β, since GARP was not expressed in M2. In contrast, the iC3b receptor CD11b, another client of GRP94, is expressed in M2 and Tg induced its decrease. iC3b induces tolerance in macrophages^25^ and we previously reported that serum GRP94 interacted with the complement C3/C3b in GvHD^4^. Here, this association was confirmed in macrophages. Interestingly the number of interactions GRP94-C3 observed by Duolink™ PLA was lower in M2 compared to M1 and increased after Tg treatment. Immunofluorescence confirmed our results by showing that, in Tg conditions, the higher number of interactions in PLA could be explained by both a higher C3/C3b level and colocalization with GRP94. We believe unlikely that C3 interacts with GRP94 in the ER, but with the alpha chain, after cleavage of pro-C3 in the Golgi apparatus, in accordance with results showing that, after Tg treatment, GRP94 enters the Golgi complex^27,34^. Although both M2 and Tg-treated M2 secreted C3, only after Tg treatment a band corresponding to C3b was observed. C3 is cleaved by cathepsin L in T lymphocytes^18^. Here we show that Tg induced cathepsin L secretion, in accordance with published results^35,36^. It is therefore likely that C3 may be mainly cleaved extracellularly by cathepsin L in Tg-treated conditions. Moreover, GRP94 was co-immunoprecipitated with C3-C3b in the supernatant confirming ELISA results and suggesting that it may remain associated to C3 during the cleavage. The most likely explanation for a chaperone like GRP94 is that through the association with C3, it affects its accessibility to cathepsin. So, we hypothesized that in Tg conditions, GRP94 is secreted by M2 together with C3 and cathepsin L and favors C3 cleavage by cathepsin-L, which could explain extracellular GRP94 pro-inflammatory role. Interestingly, the region of C3 (AA 749–954) that we previously showed to interact with GRP94^4^ is the region to which binds factor H, a C3 regulator, which acts also as a cofactor of cathepsin L (Supplementary Fig. S6)^37^. Moreover, PU-WS13 prevented Tg-induced decrease in cellular cathepsin L and PLA results indicate that Tg favors the proximity between GRP94 and cathepsin L, thus confirming a link between GRP94 and cathepsin L.

Tg treatment increased CD46 CYT1 isoforms, which play a major role in the differentiation of Th1 lymphocytes, whereas the CYT2 isoforms were mainly expressed on M2 cells. Tg-induced C3 cleavage into C3b and C3a may contribute to the pro-inflammatory profile observed. Indeed, C3b binding to CD46 CYT1 is well-known to contribute to IFNγ secretion in T cells^38^ and induces NO production in mouse macrophages expressing CYT1 but not CYT2^39^. C3a induces pSTAT1, pNFkB and TNFα production in murine macrophages and decreases M2 markers during cotreatment with IL-4^40^.

Finally, we found that iC3b was present on M2 macrophages but decreased after Tg treatment along with a decrease in CD11b. Moreover, its decrease after treatment with PU-WS13 showed that its presence depends on GRP94.

**Fig. 8:**
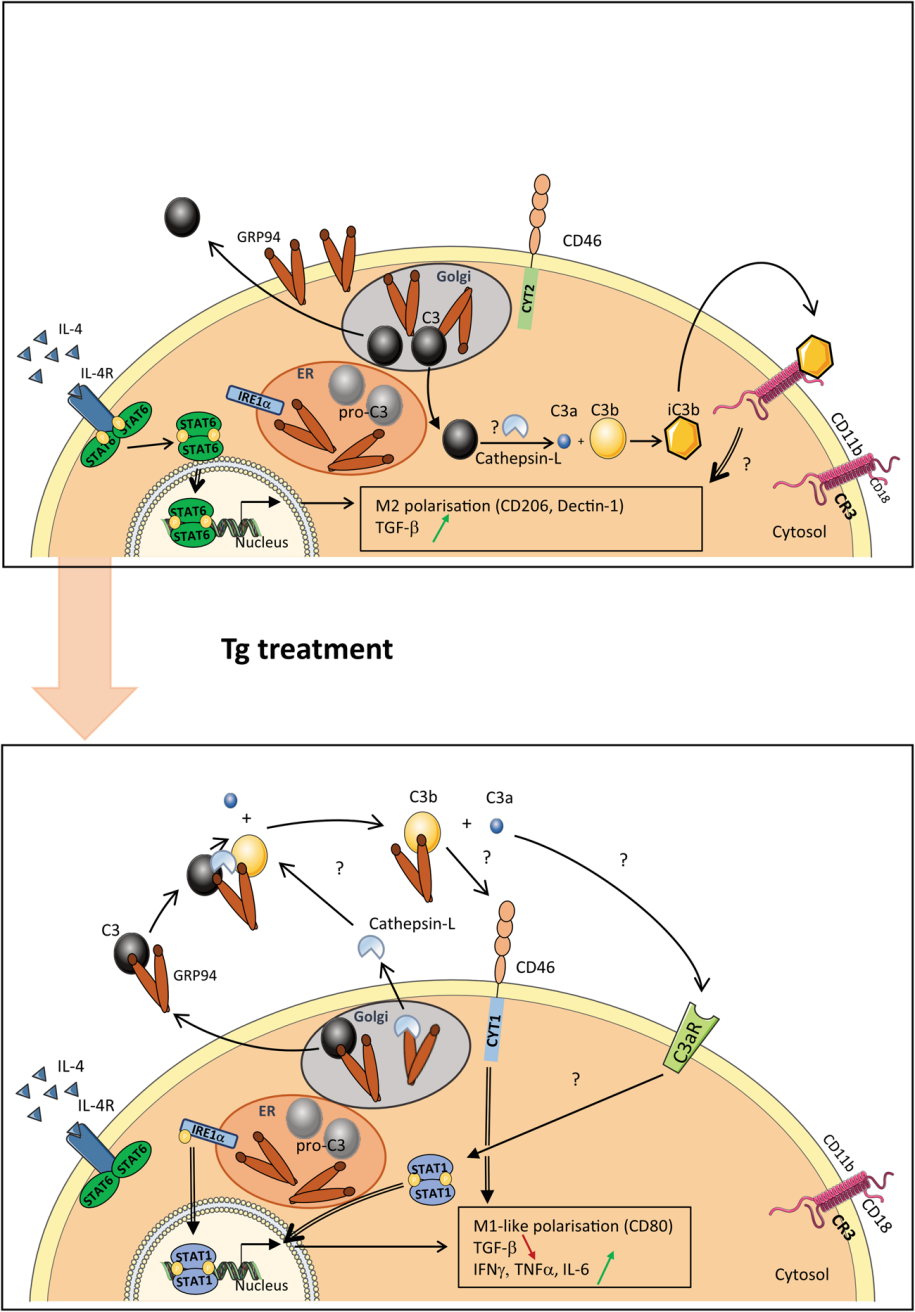
Hypothetic scheme integrating GRP94 and C3 in basal and Tg treated M2 macrophages. Upper panel: M2 macrophages are activated by IL-4/STAT6 signalization and express membrane GRP94 as well as high levels of CD11b/CD18 complement receptor CR3 and mainly CYT2 CD46 isoforms. C3, although found in the extracellular medium, is not cleaved because of the absence of extracellular cathepsin L. Within the cell, cathepsin L may cleave C3, thus generating C3b that is then inactivated in its iC3b fragment. Binding of iC3b on its receptor CR3 may contribute to the M2 phenotype. Lower panel: Under Tg-induced ER stress, the IL-4/STAT6 signalization is inhibited. GRP94 cell surface expression is highly decreased as well as CD11b and membrane-bound iC3b. Intracellular GRP94 interacts extensively with C3 and they are both secreted. Cathepsin-L is also secreted and can cleave C3 extracellularly into C3b and C3a. Both fragments may induce the pro-inflammatory profile pSTAT1+, C3b being no more inactivated and interacting with CD46 CYT1 isoform which is increased, while C3a can interact with its receptor C3aR.

In conclusion, GRP94 is expressed at the membrane of M2(IL-4) whereas it is not on Tg-treated M2 nor on M1 macrophages, suggesting that GRP94 may be a signature for M2 cells. Membrane-bound iC3b is also a marker of M2 and depends on functional GRP94. We demonstrate that Tg-induced ER stress in M2 leads to a switch towards a pro-inflammatory profile, which is both linked to IRE1α activation and dependent on a functional GRP94, and may be related to its ability to be co-secreted with C3 and facilitate its activation in the extracellular medium. Macrophages have a key role in cancer and inflammatory and autoimmune disorders and the modulation of the anti-inflammatory function of M2 macrophages represents an issue in these diseases. Our results showing that membrane GRP94 could be a marker of M2 as well as its role in modulating their anti-inflammatory function during ER stress should contribute to develop new therapeutic approaches. In Fig. 8, we propose a hypothetic model integrating GRP94 and complement C3 involvement in basal conditions and Tg-stressed M2 macrophages.

## Materials and methods

### PBMC-derived macrophages from healthy volunteers

Buffy coats were obtained from Etablissement Français du Sang and PBMC separated using Ficoll. After adhesion, monocytes were cultivated in RPMI 10% FBS with 100 ng/ml GM-CSF or M-CSF (130-093-866, 130-096-492, Miltenyi Biotec, Bergisch Gladbach, Germany) for 6 days, then respectively activated with 20 ng/ml of IFNγ (130-096-484, Miltenyi Biotec) and 100 ng/ml LPS (L2887, Sigma-Aldrich) or IL-4 (130-093-922, Miltenyi Biotec) and treated or not with Tg 25 nM (T9033, Sigma-Aldrich-Aldrich, Saint Louis, Missouri, USA), Tm 100 nM (T7765, Sigma-Aldrich) or Cch 1 μM (212385-100MG, Sigma-Aldrich) in serum free Opti-MEM for the whole activation period. In some experiments, the GRP94 inhibitor PU-WS13 (MerckMillipore, Burlington, Massachusetts, USA) or HSP90 inhibitor NVP-BEP800 (S1498-10MG Selleckchem, Houston, Texas, USA) was added 24 h prior activation.

THP1 monocytes (ATCC) were grown in RPMI 10% FBS and tested for mycoplasma contamination before use.

### Mice and tumor growth

Mice studies were conducted in accordance with the local guidelines for animal experimentation. Protocol no. 7830 was approved by ethical committee (C2EA Grand Campus Dijon). To induce tumor formation, 5 × 10^4^ triple negative breast cancer 4T1 cells were injected into the mammary fat pad of female BALB/c (7 weeks, *n* = 4) from Charles River (Wilmington, Massachusetts, USA). Experiments were performed within the ethical limitation of 1500 mm^3^. Tumors were collected and fixed in 10% formaldehyde before being paraffin embedded.

### Western blotting

Macrophages were lysed in buffer supplemented with protease inhibitors. Supernatant proteins were precipitated using methanol and chloroform. Samples were prepared in classical loading buffer and loaded on SDS-PAGE gel before being transferred on PVDF membrane. Primary antibodies were incubated overnight at 4 °C. Primary antibodies: BiP (#3177), MMP-9 (#13667 S), IRE1α (#3294 P), pIRE1α (ab124945, Abcam, Cambridge, UK), PERK (#5683 P), ATF6 (#65880 S), pIκB (#9246 S), IκB (#4812 S), pSTAT1 (#9167 S), STAT1 (#9175 S), pSTAT6 (#9361 S), STAT6 (#5397 S, Cell Signaling Technology, Danvers, Massachusetts, USA), GRP94 (9G10 Mab, ADI-SPA-850, Enzo Life Sciences, Villeurbanne, France), GARP (PA5-23301), Cathepsin-L (PA5-88413, ThermoFisher Scientific, Waltham, Massachusetts, USA), C3d (A0063, DAKO, Les Ulis, France). Secondary antibodies used for revelation were conjugated to HRP (Jackson ImmunoResearch, Cambridge, UK).

### Flow cytometry

Macrophages were washed and Fc receptors blocked prior staining for 30 min with primary antibodies or isotype controls. If necessary, a secondary conjugated Alexa-488 antibody was incubated for 30 min after washing. All data were acquired with FacsDiva^®^ software on BD FACSCanto II^®^ or BD LSR Fortessa^®^ cytometer and analyzed with FlowJo^®^ software. Antibodies: GRP94 (ADI-SPA-850-488-F, Enzo Life Sciences), CD80 (305220), GARP (352506, Biolegend, San Diego, California, USA), CD206 (551135), Dectin-1 (564471), CD18 (555923), CD11b (557918, BD Pharmingen, Franklin Lakes, New Jersey), iC3b (A209, Quidel, San Diego, California, USA), secondary antibody anti-mouse-488 (A11059, ThermoFisher Scientific).

### ELISA

TNFα, IFNγ, IL-6 and IL-1β were quantified with Multiplex technology using R&D System kit (Minneapolis, Minnesota, USA) (LXSAHM-05). Data were analyzed with Bioplex 2000™ device. TGF-β was quantified in cell culture supernatants by R&D System Quantikines.

Extracellular GRP94 was quantified using the ELISA kit from Cloud Clone.

### Co-immunoprecipitation

THP1 cells were lysed for 15 min in 50 mM NaCl, 5 mM EDTA, 10 mM glycerol-phosphate, 10 mM Tris-HCl pH = 8 buffer containing 0.5% v/v PMSF protease inhibitor. Lysates were centrifuged and incubated with anti-C3d (A0063, DAKO) or isotype control overnight at 4 °C. Magnetic protein A-conjugated beads were added and incubated for 30 min before being centrifuged, washed and resuspended in loading buffer. Samples were loaded on SDS-PAGE gel, transferred on PVDF membranes prior to be incubated with anti-GRP94 (9G10) antibody overnight, then with anti-C3d and anti-β-actin-HRP (A3854-200UL, Sigma-Aldrich).

For extracellular C3d-GRP94 co-immunoprecipitation, supernatants (1 ml) were incubated overnight with anti-C3d.

### Duolink^®^ Proximity-Ligation Assay (PLA™)

PLA™ was performed with Duolink^®^ In Situ Orange Starter Kit Mouse/Rabbit (DUO92102-1KT) following manufacturer’s protocol. The primary antibodies couples used were: anti-C3d (A0063, DAKO) and anti-GRP94 (ab210960, Abcam, Cambridge, UK), anti-C3d (A0063) and anti-HSP105 (sc-74550, SantaCruz Biotechnology, Dallas, Texas, USA), anti-GRP94 (ab210960) and anti-TLR4 (56580, NOVUS Biological, Centennial, Colorado, USA) and anti-cathepsin-L (PA5-88413, ThermoFisher Scientific, Waltham, Massachusetts, USA). Microscopy images were taken on an Axio Imager 2 (Carl Zeiss Microscopy GmbH, Jena, Germany). Images were acquired using an AxioCam MRm monochrome CCD camera (Carl Zeiss GmbH) and analyzed using ImageJ^®^ software. Spotted quantification was performed using Icy^®^ software.

### Immunofluorescence

Cells seeded on coverslips were fixed with a 3% formaldehyde solution for 15 min at RT, washed and quenched with 50 mM NH_4_Cl for 10 min before being permeabilized with PBS-0.1% TRITON X-100 for 10 min at RT and blocked for 1 h with PBS-1% BSA FcR Blocking Reagent (130-059-901, Miltenyi). Cells were incubated with primary antibody at 4 °C overnight: anti-C3d (A0063, DAKO), anti-GRP94 (ab210960, Abcam) or anti-GM130 (AF8199, R&D System), washed and stained with secondary fluorescent antibodies. Images were acquired as described above and analyzed by Zen 2.3 Lite^®^ software.

### Immunohistochemistry

Paraffin-embedded 4T1 tumor sections were dewaxed and antigen retrieval step performed in 10 mM citrate buffer in 95 °C water bath for 30 min. After 30 min at RT, slides were incubated in PBS-Tween 0.1%, 8% BSA for 1 h, then incubated overnight at 4 °C with primary antibodies: rat anti-GRP94 (LS-B3418-50) and rabbit anti-CD206 (LS-B9805-200, Clinisciences, Nanterre, France). Tissues were washed in PBS-Tween and incubated 1 h at RT with the following antibodies anti-rat Alexa 488 (Abcam, ab150153), anti-rabbit Alexa 568 (Abcam, ab175470). Microscopy images were acquired and analyzed as described for immunofluorescence.

### RT-PCR

Total RNA was extracted with Trizol (Life Technologies) and reverse transcribed with M-Maxima™ First Strand cDNA Synthesis Kit (ThermoFisher). PCR was performed with Taq’Ozyme Purple Mix 2 (Ozyme, Saint-Cyr-l’Ecole, France) (OZYA007-200XL). The sequence of primers for CD46 were: F: 5’-GTGGTCAAATGTCGATTTCCAGTAGTCG-3’, R: 5’-CAAGCCACATTGCAATATTAGGTAAGCCACA-3’^41^.

### Cytotoxicity assay

Cytotoxicity was assessed using the CellTiter 96^®^ AQueous One Solution Cell Proliferation Assay (MTS) (G3580, Promega, Madison, Wisconsin, USA).

### Statistical analysis

Data were analyzed using GraphPad Prism software 7.03 (La Jolla, CA, USA). Normal distribution was determined using Shapiro–Wilk’s test and equal variance by Brown Forsythe’s test. Data for multiple group comparisons were analyzed by one-way ANOVA followed by Tukey or Dunnett’s post-hoc analysis. The Kruskal–Wallis test was used for multiple group comparisons that did not comply with normal homogeneity or homogeneity of variance. Two-tailed Student’s *t*-test was used to compare 2 groups. Data are expressed as mean ± s.e.m. (standard error of the mean). Details for box plots are specified in the figure legend. A *p*-value of less than 0.05 was considered statistically significant.

## Supplementary information

Supplemental figures legends

Supplemental Figure 1

Supplemental Figure 2

Supplemental Figure 3

Supplemental Figure 4

Supplemental Figure 5

Supplemental Figure 6
