# Supplementary material for: The HSP GRP94 interacts with macrophage intracellular complement C3 and impacts M2 profile during ER stress
Source: Cell Death Dis. 2021 Jan 22;12(1):114. doi: 10.1038/s41419-020-03288-x (PMC7822929; doi:10.1038/s41419-020-03288-x)
Supplement: Supplementary file 1 — Supplemental figures legends [file 41419_2020_3288_MOESM1_ESM.docx]

**Supplemental Material**

**Legends to figures**

**Figure S1: GRP94 expression during macrophage differentiation in basal and ER stress conditions**

**(A**) Western-blot analysis of GRP94 in PBMC and M1 or M2 macrophages during their differentiation at 6h and 12h after treatment with Tg (25 nM) or Tm (100 nM) **(B)** FACS analysis of GRP94 membrane expression during differentiation of M1 and M2 **(C)** FACS analysis of GRP94 membrane expression during differentiation of M2 macrophages treated or not with Tg or Tm.

**Figure S2: UPR response in macrophages treated by Tg, Tm or carbachol**

**(A)** Cell viability quantification of M1 and M2 macrophages treated with different concentrations of Tg and Tm for 72h **(B)** Western- blot analysis of the UPR sensors IRE1α and PERK at 6h and 12h in M1 or M2 macrophages treated or not with Tg or Tm **(C)** Western- blot analysis of the UPR sensors IRE1α and PERK in macrophages treated or not with Tg, Tm or carbachol (1 μM) for 24h **(D)** CD206 and CD80 markers membrane expression on M2 macrophages treated or not with Tg, Tm or carbachol (1 μM) for 48h.

**Figure S3: PU-WS13 impact on macrophages under basal or Tg-treated condition**

**(A)** ER stress induction of BiP was determined by western-blotting in Tg and/or PU-WS13 treated M1 and M2 **(B)** Cytotoxicity analysis of different doses of PU-WS13 on M1 (left panel) and M2 macrophages (right panel) **(C)** FACS analysis of membrane GRP94 on M2 macrophages treated or not with PU-WS13. **(D)** FACS analysis of CD206 and CD80 membrane expression on M2 macrophages treated or not with Tg (25 nM) and PU-WS13 (1, 5 and 25 μM) or NVP-BEP800 (50 and 500 nM) **(E)** Cytotoxicity analysis of different doses of NVP-BEP800 on M2 macrophages.

**Figure S4: Membrane GARP expression on M1 macrophages under Tg and PU-WS13 treatment**

FACS analysis of Tg-induced GARP membrane expression on M1 macrophages after treatment with different doses of PU-WS13.

**Figure S5: GRP94 expression in CD206+ cells in murine triple negative breast 4T1 tumors**

Immunofluorescence analysis of GRP94 in CD206+ cells in 4T1 mice tumors, representative images (n= 4 mice). Upper panel, magnification=x 20. Lower panel, magnification x 40 of upper panel areas. Percentage of CD206+ cells expressing GRP94 using ImageJ software for the 4 mice analyzed: 44,05% ± 8,17%.

**Figure S6: *Scheme of the C3 alpha chain 749-954 region interacting with GRP94 (*Seignez et al., 2017)**
