# Supplementary figures and images for: The HSP GRP94 interacts with macrophage intracellular complement C3 and impacts M2 profile during ER stress
Source: Cell Death Dis. 2021 Jan 22;12(1):114. doi: 10.1038/s41419-020-03288-x (PMC7822929; doi:10.1038/s41419-020-03288-x)

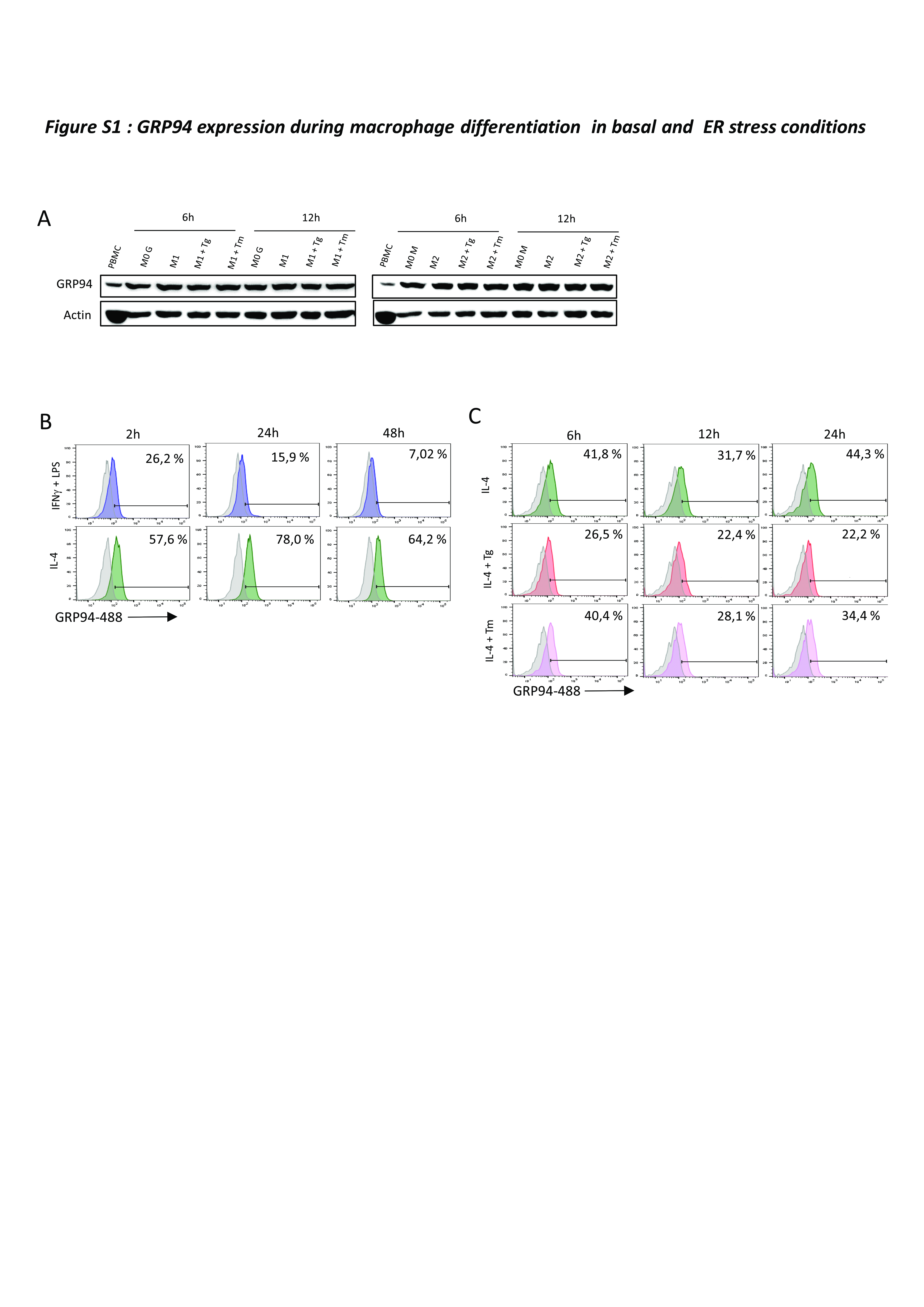

Supplement: Supplementary file 2 — Supplemental Figure 1 [file 41419_2020_3288_MOESM2_ESM.tif]

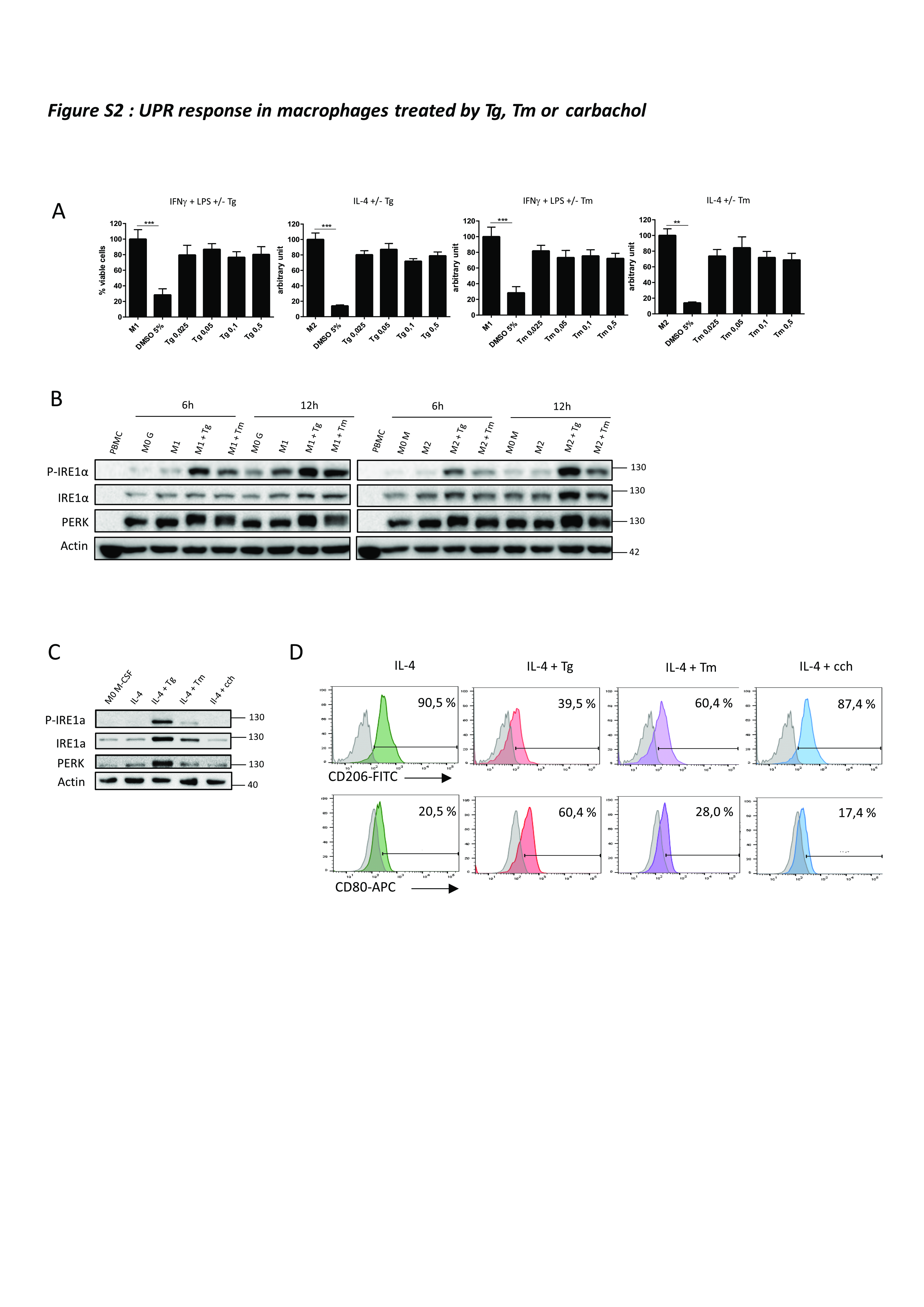

Supplement: Supplementary file 3 — Supplemental Figure 2 [file 41419_2020_3288_MOESM3_ESM.tif]

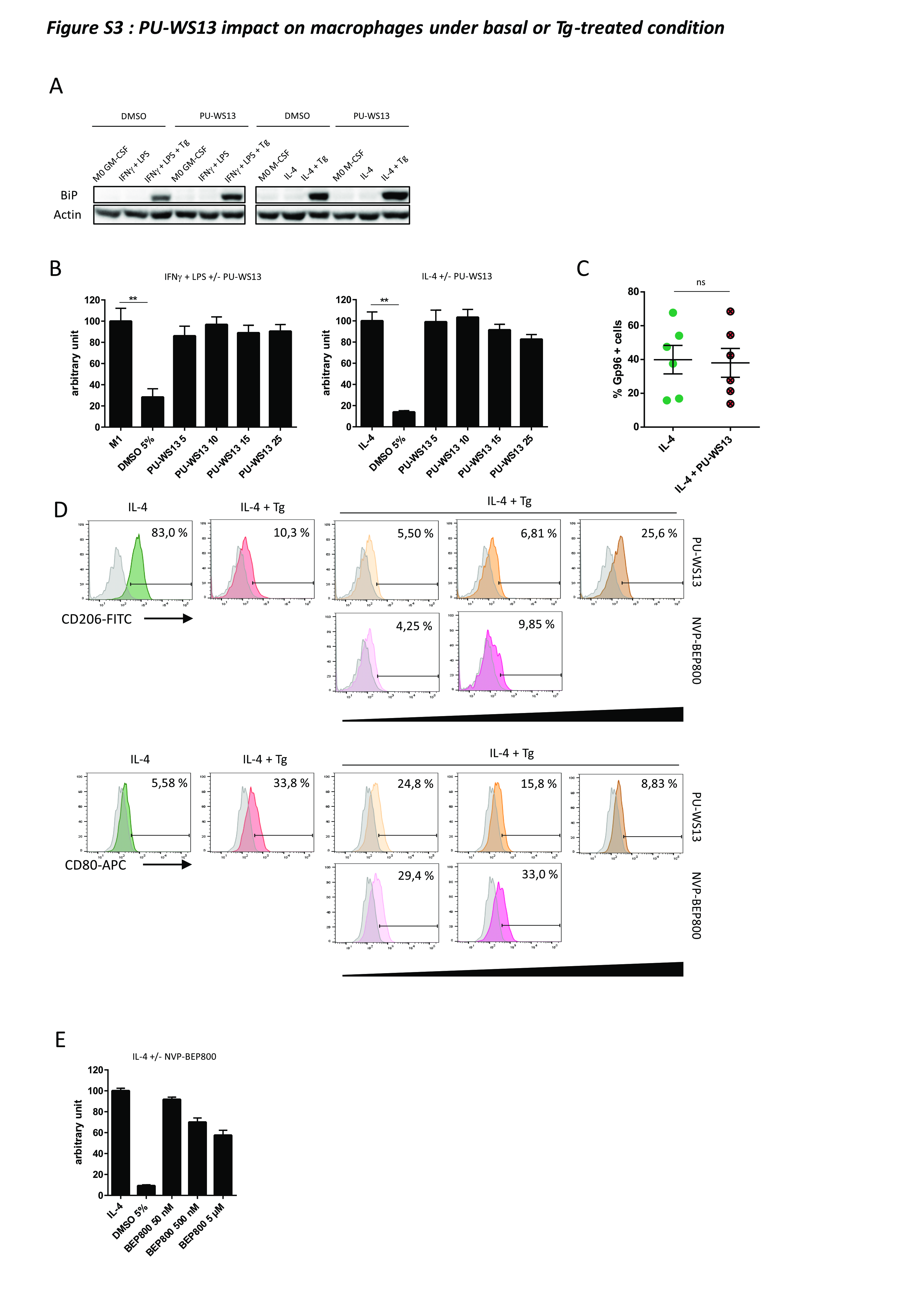

Supplement: Supplementary file 4 — Supplemental Figure 3 [file 41419_2020_3288_MOESM4_ESM.tif]

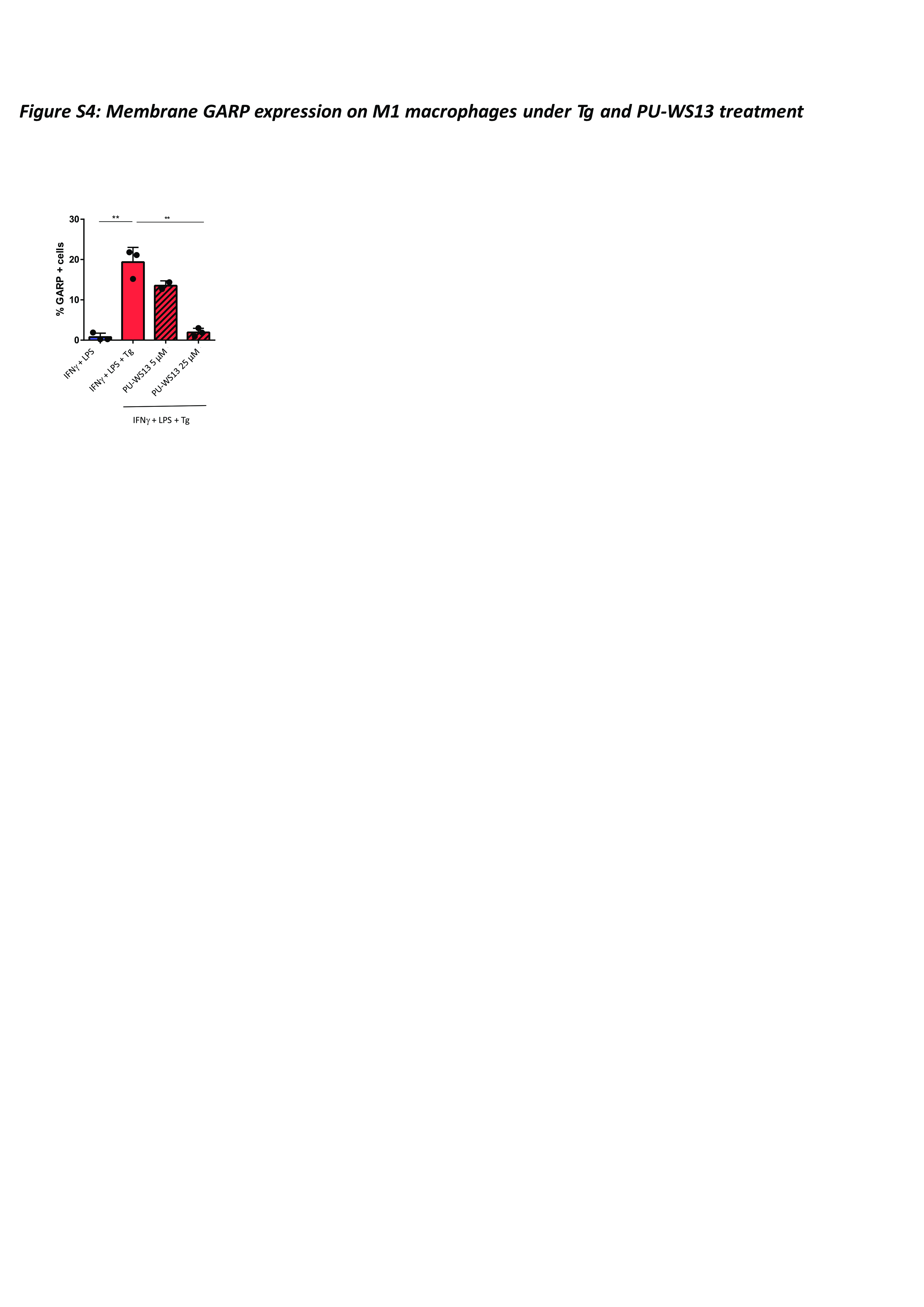

Supplement: Supplementary file 5 — Supplemental Figure 4 [file 41419_2020_3288_MOESM5_ESM.tif]

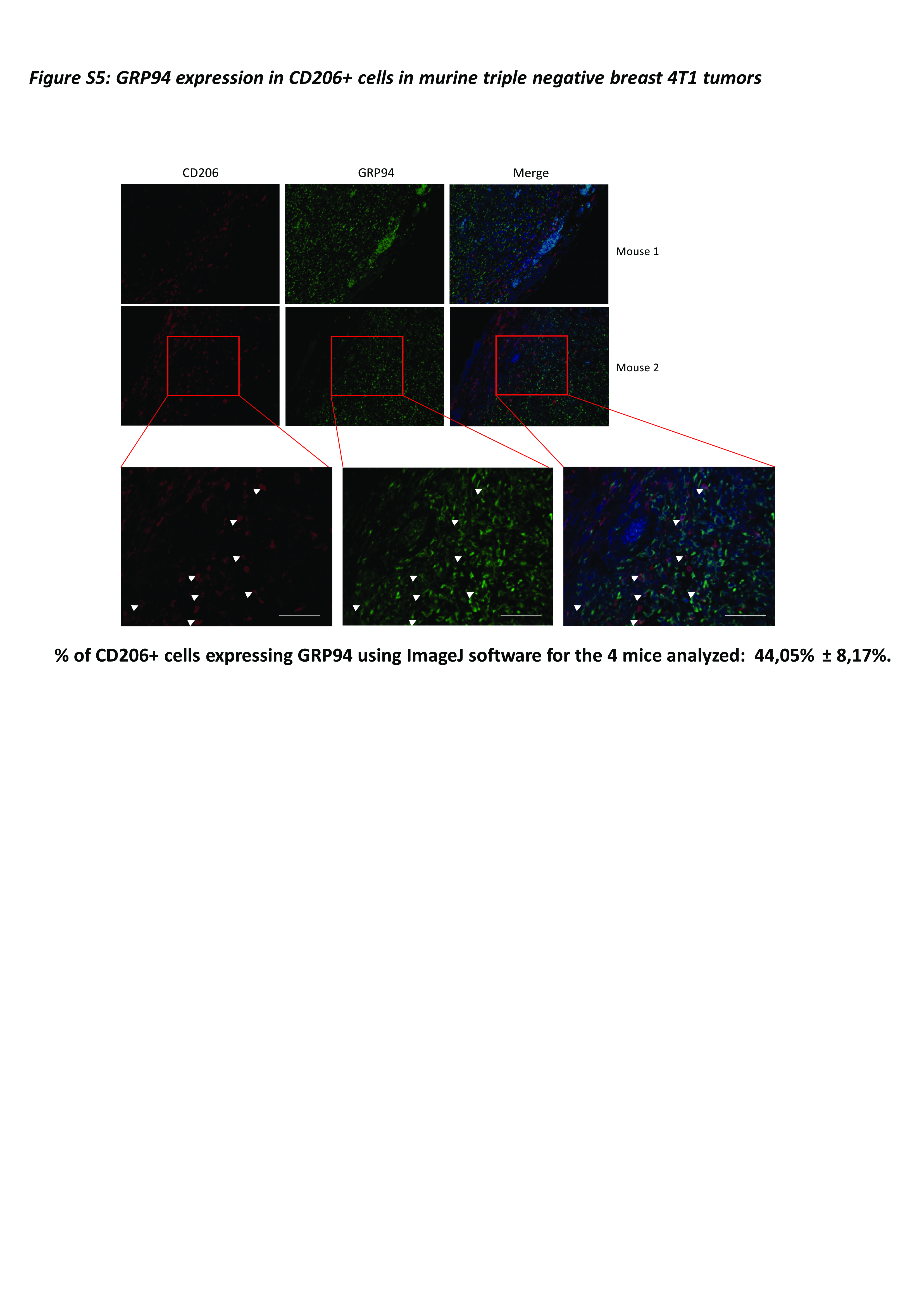

Supplement: Supplementary file 6 — Supplemental Figure 5 [file 41419_2020_3288_MOESM6_ESM.tif]

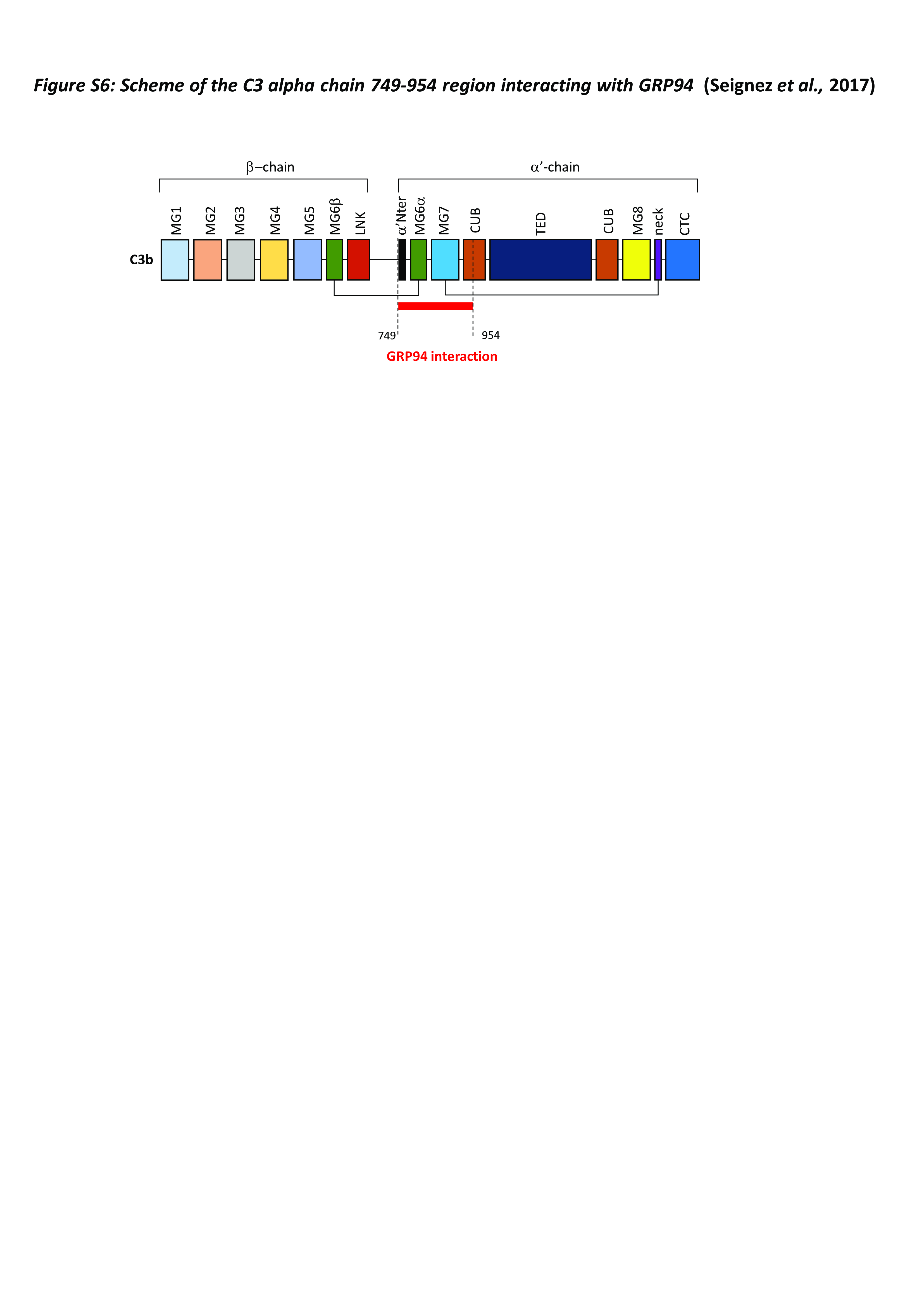

Supplement: Supplementary file 7 — Supplemental Figure 6 [file 41419_2020_3288_MOESM7_ESM.tif]
